# Modulating pyrimidine ribonucleotide levels for the treatment of cancer

**DOI:** 10.1186/s40170-020-00218-5

**Published:** 2020-10-04

**Authors:** Tanzina Mollick, Sonia Laín

**Affiliations:** 1 Department of Microbiology, Tumor and Cell Biology, Karolinska Institutet, Solnavägen 9, SE-171 65, Solna, Stockholm, Sweden; 2 SciLifeLab, Department of Microbiology, Tumor and Cell Biology, Karolinska Institutet, Tomtebodavägen 23, SE-171 65, Solna, Stockholm, Sweden

**Keywords:** Pyrimidine ribonucleotide metabolism, Cancer therapy, CAD, DHODH, UMPS, Nucleoside transporters, CDA, CTPS, Therapeutic index

## Abstract

By providing the necessary building blocks for nucleic acids and precursors for cell membrane synthesis, pyrimidine ribonucleotides are essential for cell growth and proliferation. Therefore, depleting pyrimidine ribonucleotide pools has long been considered as a strategy to reduce cancer cell growth. Here, we review the pharmacological approaches that have been employed to modulate pyrimidine ribonucleotide synthesis and degradation routes and discuss their potential use in cancer therapy. New developments in the treatment of myeloid malignancies with inhibitors of pyrimidine ribonucleotide synthesis justify revisiting the literature as well as discussing whether targeting this metabolic pathway can be effective and sufficiently selective for cancer cells to warrant an acceptable therapeutic index in patients.

## Background

Pyrimidine ribonucleotides are involved in multiple cellular processes that maintain cell growth and metabolism [1]. Aside from being the building blocks of RNA and precursors for deoxyribonucleotides, pyrimidine ribonucleotides are necessary for glycogen and cell membrane precursor synthesis, glycosylation of proteins and lipids, and in detoxification processes like glucuronidation [1–4]. In addition, uracil nucleotides can interact with G protein-coupled nucleotide receptors to activate the phosphatidylinositol-calcium second messenger system [5, 6], and cCMP as well as cUMP can themselves act as second messengers [7, 8].

The activity of enzymes involved in pyrimidine ribonucleotide synthesis is required for cellular proliferation [9–14], and it has been observed that many tumors show upregulation of these enzymes [15–20]. Thus, depleting pyrimidine ribonucleotide pools has long been considered an option for cancer treatment. In light of this, a number of inhibitors of enzymes of the pyrimidine ribonucleotide synthesis pathway have been developed in the past decades. However, due to unsatisfactory results in the clinic, further work in this line of therapy was not given priority [21–31]. It was not until recently that new insights have once again drawn attention towards pyrimidine metabolism [32–34]. In this review, we focus on the enzymes in the pyrimidine synthesis and degradation pathways for which small molecule inhibitors are available and either considered or evaluated in clinical trials.

## Pyrimidine ribonucleotide synthesis

**Fig. 1:**
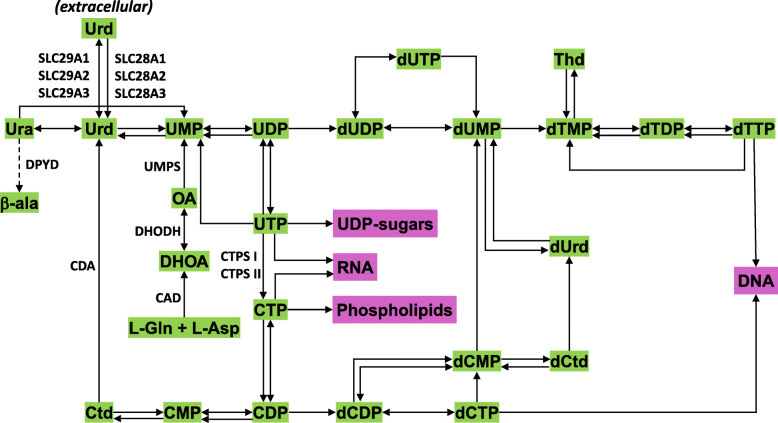
Simplified schematic of pyrimidine nucleotide synthesis showing enzymes targeted for cancer therapy. For details, see the KEGG pathway, and for abbreviations, refer to the list

Cellular pyrimidine ribonucleotide pools are maintained through the de novo synthesis and salvage pathways [1, 4] (Fig. 1). The relative importance of these pathways depends on the cell type and its physiological state. It has been suggested that rapidly proliferating cells, whether normal (e.g., activated T cells) or cancerous, depend on the de novo pyrimidine pathway, whereas differentiated cells, having lower demands, usually rely on the less energy requiring pyrimidine salvage pathway [1].

## The de novo pyrimidine ribonucleotide synthesis pathway

In the de novo synthesis pathway, the pyrimidine ring structure is assembled through a multistep pathway with l-glutamine and l-aspartate as precursors [1, 2, 4]. Whereas l-aspartate is a non-essential amino acid, l-glutamine is designated as a conditionally essential amino acid that becomes vital during periods of rapid growth or disease [35]. These two precursors are transformed into dihydroorotate by the three activities of the multifunctional enzyme CAD (carbamoylphosphate synthetase II, aspartate transcarbamoylase, and dihydroorotase). Next, orotate is formed by the action of dihydroorotate dehydrogenase (DHODH). DHODH is the only enzyme in the pathway located in mitochondria. DHODH catalyzes the oxidation of dihydroorotate to orotate using ubiquinone as an electron acceptor. Therefore, DHODH is dependent on and contributes to the activity of the mitochondrial electron transport chain [1, 2, 4]. The third enzyme of the de novo synthesis pathway is the bifunctional uridine monophosphate synthetase (UMPS) which catalyzes the formation of the first ribonucleotide product uridine 5′-monophosphate (UMP) through the action of its orotate phosphoribosyltransferase and orotidine-5′-monophosphate decarboxylase activities.

Once UMP is formed, further steps in the anabolic pathway result in the formation of uridine 5′-diphosphate (UDP) and uridine 5′-triphosphate (UTP). UTP can be used for protein glycosylation and glycogen synthesis through the formation of UDP-linked sugars. It is also the precursor for cytidine 5′-triphosphate (CTP) through the action of CTP synthetases I and II (CTPS I and II) [1, 4, 36, 37]. Most importantly, this is the only path to obtain cytosine nucleotides de novo in mammals. Aside from being a building block in RNA, CTP can be converted to cytidine 5′-diphosphate (CDP), which in turn can be transformed into deoxyCDP (dCDP) by ribonucleotide reductase (RNR) to provide building blocks for DNA. CTP is also essential for membrane formation, which relies on CDP-linked phospholipid precursors [37, 38].

Like CDP, UDP can be converted into dUDP by RNR. In turn, dUDP can be dephosphorylated into dUMP, a precursor for dTTP (2′-deoxythymidine-5′-triphosphate), and used for DNA synthesis. dUMP can also be phosphorylated into dUTP and incorporate in DNA, causing activation of the DNA damage response [39]. Excessive repair events can increase the risk of DNA fragmentation and cause cell death or genomic instability. Therefore, an excess of dUTP might contribute to the appearance of mutations that arise randomly during DNA replication [40].

## Salvage pathways

A fundamental difference between purine and pyrimidine ribonucleotide salvage pathways is that purine ribonucleotides are recycled from their bases whereas pyrimidine ribonucleotides are mainly salvaged from their nucleosides [36]. Accordingly, in patients with deficient de novo pyrimidine ribonucleotide synthesis, uridine (but not uracil) is able to overcome pathological manifestations [41]. The precursors in the salvage pathway include intracellular uridine and cytidine as well as pyrimidine nucleosides from extracellular fluids [3]. In addition, it can be envisaged that pyrimidine ribonucleotides can be recycled from UDP-linked sugars, CDP-linked phospholipid precursors, pyrimidine deoxyribonucleotides, and RNA.

Most of the data on pyrimidine ribonucleoside/ribonucleotide levels in the Human Metabolome Database is derived from a review written in 1994 [3]. In a more recent study on plasma from healthy individuals [42], the following average values were obtained: cytidine (0.25 μM), uracil (2.10 μM), uridine (3.12 μM), and orotate (0.89 μM). Cytosine was below the detection limit of the method. Strikingly, there are no enzymes that can lead to or process free cytosine in mammals (see KEGG pathways). One potential difference between mice and humans is the concentration of cytidine in plasma. According to the current data [3], cytidine levels in the plasma of rodents are higher than in human plasma. It is likely that this feature may cause differences between mice and humans in response to drugs affecting ribonucleotide pools.

## Pyrimidine nucleoside/nucleobase transporters

Extracellular uridine is imported into cells by two classes of pyrimidine and purine nucleoside/nucleobase transporters: equilibrative and concentrative [43, 44]. These transporters are also involved in the uptake of anticancer nucleoside analogues such as cytarabine and gemcitabine [45]. Furthermore, single nucleotide polymorphisms in drug transporters may contribute to variations between individuals in response to nucleoside drugs [46, 47].

Equilibrative nucleoside transporters (ENT1, ENT2, ENT3, and ENT4, also known as SLC29A1, SLC29A2, SLC29A3, and SLC29A4) are bidirectional sodium-independent transporters [43, 44, 47, 48]. Human ENT1 and ENT2 transport purine and pyrimidine nucleosides, while ENT2 and ENT3 also transport nucleobases [43, 48]. ENT4 is uniquely selective for adenosine and a variety of organic cations, despite its structural similarity with other ENTs [49]. With regard to cellular localization, ENT1, ENT2, and ENT4 are primarily located in the cytoplasmic membrane. However, ENT1 and ENT2 can additionally be found in the nuclear membranes and, in case of ENT2, also in the mitochondrial membrane. ENT3 only appears to function in intracellular membranes including those of the lysosomes and mitochondria [43]. Concentrative nucleoside transporters (CNT1, CNT2, and CNT3, also known as SLC28A1, SLC28A2, and SLC28A3) are unidirectional sodium-dependent active transporters that have higher affinity for uridine than ENTs [43, 48, 50] but a lower turnover rate of transport [43, 51]. Although all the CNTs transport uridine, CNT1 preferentially transports pyrimidine nucleosides, CNT2 purine nucleosides, and CNT3 both pyrimidine and purine nucleosides [43, 48]. CNT1–3 are primarily located in the cytoplasmic membrane, but CNT3 is additionally present in subcellular membranes of specific cell types [43]. Both ENTs and CNTs participate in maintaining uridine (as well as other nucleoside and nucleobase) homeostasis through their activities in the intestine, liver, and kidneys [43, 50].

## Modulation of uridine plasma levels

Uridine plasma levels should be taken into consideration when assessing the response to drugs lowering pyrimidine ribonucleotide pools. It is generally accepted that the liver plays a central role in maintaining plasma uridine by synthesizing and degrading uridine [36]. In addition, blood platelets contain high levels of UTP and may constitute another source of uridine [52]. Erythrocytes, which can rapidly take up orotate and convert it to UDP-glucose, are also thought to play an important role as uridine reservoirs [53]. In fact, uridine and glucose can be supplied to the brain, skeletal muscles, and peripheral tissues by the catabolism of erythrocyte UDP-glucose [36]. A recent study on mice, rats, and humans reported that fasting increases plasma uridine through a mechanism that involves uridine biosynthesis by adipocytes, whereas, in the postprandial state, liver-induced bile excretion results in plasma uridine clearance [54]. In support of this study, longer starvation times in human volunteers led to even more prominent increases in uridine plasma levels that were accompanied by a smaller rise in plasma cytidine and CTP [55]. Further confirmation came from a study where uridine as well as uracil and dihydrouracil levels in human plasma were observed to drop after food intake [56] and were also seen to be affected by circadian rhythms [56, 57]. In agreement with these observations, it has been seen that the oral bioavailability of uridine is low (5.8–9.9%), due to poor absorption through the gastrointestinal mucosa [58]. Efforts at increasing the oral bioavailability of uridine led to the discovery of a prodrug, PN-401, which allowed sustained plasma levels of > 50 μM [59]. Altogether, these studies may suggest that drugs lowering de novo pyrimidine ribonucleotide synthesis may be more effective when given after meals.

On a different note, an interesting study by Steculorum et al. may show a possible link as to how uridine plasma levels may affect feeding patterns [60]. They reported that high UDP levels in the hypothalamus were related to increased feeding behavior in mice through the activation of P2Y6 purinergic receptors in the agouti-related peptide neurons. Moreover, increasing plasma uridine levels, through intraperitoneal injection, promoted this activity by the increased synthesis of UDP in the brain. Interestingly, in metabolic disorders like obesity and type 2 diabetes mellitus, high uridine levels are sustained in the plasma which in turn affects food intake behavior due to high UDP levels in the brain [60]. This could indicate that the efficacy of inhibitors of de novo pyrimidine ribonucleotide synthesis may be affected in comorbid conditions that include such metabolic disorders.

## Pyrimidine ribonucleotide degradation

Based on the pyrimidine metabolic pathway provided in the Kyoto Encyclopedia of Genes and Genomes database (KEGG pathway), UMP degradation occurs through the action of 5′-nucleotidases and uridine phosphorylases 1 and 2, which transform UMP into uridine and uridine into uracil, respectively. Uracil is finally transformed into β-alanine by the action of the enzymes dihydropyrimidine dehydrogenase (DPYD), dihydropyrimidinase, and β-ureidopropionase. This last enzyme leads to the synthesis of β-alanine through an irreversible reaction.

In humans, cytidine 5′-monophosphate (CMP) can only be transformed into cytidine but not to cytosine. Cytidine can only be converted into uridine by cytidine deaminase (CDA). dCMP and dUMP can both be converted into uracil whereas dTMP, after its conversion into thymidine and thymine, enters valine, leucine, and isoleucine metabolism (see the KEGG PATHWAY Database—GenomeNet).

Differences in the activity of the enzymes involved in pyrimidine ribonucleotide degradation are essential to predict 5-fluorouracil, gemcitabine, and cytarabine toxicity in cancer patients. These differences may be due to single nucleotide polymorphisms (SNPs) in CDA [61, 62] or DPYD [63] and, as shown for DPYD, related to food intake [56].

## Regulation of pyrimidine ribonucleotide synthesis enzymes: CAD

Studies on the regulation of pyrimidine ribonucleotide synthesis are available only for a few of the enzymes in the pathway and mainly refer to the trifunctional enzyme CAD, the first enzyme in the de novo pathway [37, 64]. Below, we discuss the key known factors involved in the regulation of CAD, many of which can be overactive in cancers. The reason we summarize these studies here is because pharmacologic modulation of these factors should affect CAD and therefore pyrimidine ribonucleotide synthesis.

### Regulation of enzyme expression

The promoter of the CAD gene has E-box sequences for c-myc binding, and c-myc clearly increases CAD expression [65, 66]. Furthermore, of the three enzymes in the de novo UMP synthesis pathway, CAD is the one that is more frequently overexpressed in tumors (FireBrowse database). DHODH as well as CTPS and enzymes of the purine synthesis pathway are also increased upon c-myc expression in Burkitt lymphoma cells [67]. Estrogen receptor/Sp1 complexes are also positive modulators of CAD transcription [68] whereas HIF1α negatively regulates CAD expression [69].

### Allosteric regulation

The carbamoylphosphate synthetase II (CPSII) domain of CAD is allosterically activated by ATP and phosphoribosyl pyrophosphate (PRPP) as well as inhibited by uracil and cytosine nucleosides UMP, UDP, UTP, CTP, and UDP-glucose [37, 70–72]. The activation of CAD (as well as of UMPS) by PRPP provides a link between the purine and pyrimidine synthesis pathways [37, 70–72].

### Post-translational modifications

CAD regulation can also be mediated through phosphorylation by the mitogen-activated protein kinase cascade (MAPK, also referred to as Ras-Raf-MEK-ERK cascade) and cyclic adenosine monophosphate-dependent protein kinase (PKA) cascades [37, 73]. It has been demonstrated, both in vitro and in vivo, that in the presence of a growth stimulus, ERK2 phosphorylates CAD at Thr456 and also alters the allosteric regulation of CAD, whereby its activation by PRPP is promoted and feedback inhibition by UTP is suppressed [37, 73, 74]. The MAPK-mediated activation of CAD can be antagonized by PKA phosphorylation of its serine residue at 1406. This leads to the reversal of the sensitivity of CAD to PRPP and UTP, consequently downregulating ribonucleotide synthesis [73, 75, 76]. The sequential coordination of MAPK and PKA phosphorylation of CAD has been closely associated with the cell cycle [73, 77]. MAPK phosphorylation of CAD occurs in cells entering early S phase, when the need for pyrimidine ribonucleotides is greatest, whereas PKA phosphorylation predominates at late S phase [77]. In addition, it has been reported that phosphorylation of CAD at Ser1873 by protein kinase C (PKC) may precede activation of CAD by the MAPK cascade [78].

It is not surprising that cancer cells would take advantage of these regulatory mechanisms to increase cell proliferation. For example, in the MCF7 breast cancer cell line, pyrimidine ribonucleotide synthesis goes unchecked due to CAD overexpression together with continuous phosphorylation at the MAPK site and absence of phosphorylation at the PKA site [79]. The MAPK pathway is frequently dysregulated in cancer due to mutations or amplifications in the upstream components of the pathway, which eventually lead to ERK hyperactivity [80–83]. Thus, targeting the pyrimidine ribonucleotide synthesis pathway may at least in part weaken the tumor promoting effect of alterations in the MAPK cascade.

Another protein kinase that has been shown to regulate CAD is mechanistic (or mammalian) target of rapamycin complex 1 (mTORC1) [84, 85]. mTORC1 is one of the two catalytic subunits of mTOR, a key kinase in balancing anabolic and catabolic processes to promote cell growth by stimulating glutamine metabolism and blocking autophagy [86]. It can also promote protein synthesis and enhance lipid synthesis, and there is emerging evidence linking mTORC1 activity to nucleotide metabolism [84, 85, 87, 88]. In 2013, phosphoproteomics and metabolomics profiling studies revealed that mTORC1 could stimulate pyrimidine ribonucleotide synthesis through activation of its downstream target S6 kinase 1, a kinase that phosphorylates the Ser1859 residue in CAD [84, 85] and activates the dihydroorotase domain of CAD [85]. Furthermore, phosphorylation at the Ser1859 site results in the oligomerization of CAD and enables steric channeling of substrates [84]. Interestingly, an upstream effector of mTORC1, Rheb, which is a small GTPase that belongs to the RAS superfamily, has also been shown to directly bind to CAD and regulate its activity [89].

mTORC1 signaling occurs downstream of the MAPK cascade and the phosphoinositide 3-kinase (PI3K)/AKT pathway. These pathways converge at mTORC1 by phosphorylating and inhibiting tuberous sclerosis complex 2 (TSC2), which is a negative regulator of mTORC1 [87, 88, 90]. Any mutation or amplification of the upstream effectors of mTORC1 or loss of function of the tumor suppressors that inhibit mTORC1, like TSC2, could lead to overstimulation of mTORC1 and drive tumor progression [86, 90, 91]. In fact, a majority of human tumors have been shown to possess mTORC1 hyperactivity and this has led to an increased interest in the development of more effective inhibitors than rapamycin [92]. However, mTORC1 inhibitors have shown unpredictable side effects in the clinic [93]. Furthermore, mutations of mTOR and activation of alternate proliferation pathways have been observed in response to mTORC1 inhibitors [86]. As mentioned above for MAPK inhibitors, perhaps, inhibitors of the pyrimidine ribonucleotide synthesis pathway may at least partially contribute to eliminate mTORC1 inhibitor-resistant clones.

## Targeting enzymes involved in pyrimidine ribonucleotide metabolism for the treatment of cancer

One important aspect to consider in any therapeutic approach is genotoxicity. And an important safety issue that needs to be addressed when using agents that deplete ribonucleotide pools is whether they harm the genome, and if they do whether this DNA damage is limited to cancer cells. In 1996, the group of Geoffrey Wahl [94] reported that inhibition of de novo ribonucleotide synthesis has different consequences depending on p53 status and that normal cells primarily respond by arresting in the G1 phase of the cell cycle. One interpretation of these results is that in normal cells, which have fully functional cell cycle checkpoints, a reduction in ribonucleotide levels could act as a warning signal that activates the cell cycle arresting function of p53 rather than its pro-apoptotic function. An arrest in G1 can be reversible, and therefore, normal cells may recover from ribonucleotide depletion. Thus, it is possible that inhibiting ribonucleotide synthesis is non-genotoxic to normal cells and causes reversible cell cycle arrest. Whether this holds true for highly proliferating cells such as activated T cells needs to be investigated further. Indeed, inhibitors of pyrimidine ribonucleotide synthesis such as leflunomide are used as immunosuppressants [95, 96]. This is of importance in the context of cancer as it may debilitate the anti-oncogenic effects of the immune system.

Cancer cells frequently have defective G1/S checkpoints and are therefore less likely to arrest in G1 than normal cells upon stress. If cells enter S phase without sufficient nucleotide pools, stalled replication forks may appear, ultimately leading to extensive DNA damage. Whether this leads to cell death may depend on the presence of intact TP53. For cancer cells that retain wild-type p53, there is evidence suggesting that they will accumulate in S phase with high levels of active p53 and rapidly die [33]. Activation of p53 may be more likely to promote cell death in S phase cells than in cells in G1 (see below). Cancer cells defective for p53 may also accumulate in S phase upon ribonucleotide depletion and subsequent deoxyribonucleotide pool depletion, but what happens to these cells at this vulnerable stage is still unclear. In the case of p53-deficient acute myeloid leukemia cells for example, inhibition of the pyrimidine ribonucleotide de novo synthesis enzyme DHODH has been shown to cause differentiation and death [97].

Altogether, it is possible that inhibitors of pyrimidine ribonucleotide synthesis may help to control cancer progression without causing irreparable damage to normal tissue. Whether efficacy will be reached in the clinic is still an open question that will hopefully be answered soon by ongoing clinical trials. Below, we focus on efforts to modulate the function of enzymes and transporters involved in pyrimidine ribonucleotide metabolism and discuss their potential for cancer therapy.

## CAD inhibitors

PALA (*N*-phosphonacetyl-l-aspartate), an inhibitor of the aspartate transcarbamoylase activity of CAD, was tested in cancer patients in the 1980s but did not make it beyond a phase II trial [26–31, 98]. In addition to its lack of efficacy, PALA was shown to cause DNA damage even to normal cells [99]. Whether this is due to inhibition of CAD is unknown as PALA may not be specific and, for example, PALA is a potent inhibitor of human carbonic anhydrase IV [100]. To our knowledge, various modifications of PALA have not yet rendered more effective inhibitors [101] although encapsulating PALA into liposomes may improve delivery and efficacy according to tests in mice [102, 103].

Thus, it is unclear as to whether CAD is not sufficiently inhibited by PALA and its analogues, or if this enzyme is not a suitable target for cancer treatment. Since CAD is a trifunctional enzyme, it is possible that blocking other enzymatic activities aside from the aspartate transcarbamoylase function may constitute a better option.

## DHODH inhibitors

Mutations in DHODH cause postaxial acrofacial dysostosis or Miller syndrome [104], a rare condition with distinctive craniofacial malformations that occur in association with limb abnormalities but not with cognitive or growth problems. The DHODH inhibitor leflunomide and its active metabolite teriflunomide have long been used for chronic diseases such as rheumatoid arthritis and multiple sclerosis [95, 96]. Thus, this suggests that targeting DHODH for cancer therapy is likely to be a safe approach. The next most advanced DHODH inhibitor with regard to clinical testing is ASLAN003 which has undergone a phase I study (https://clinicaltrials.gov/). Although the results from a phase I study with this compound are not published, ASLAN003 is now in phase II for the treatment of acute myeloid leukemia.

In the 1980s, brequinar, which still ranks as one of the most potent and selective DHODH inhibitors, was shown to work in mouse models for solid tumors as well as in a leukemia murine model [105]. Even leflunomide and teriflunomide, although weak and non-specific against DHODH, have proven efficacious in animal models [106–108]. Later on, in the early 1990s, brequinar was tested against solid tumors in patients [21–25]. Unfortunately, these trials did not demonstrate efficacy below the maximum tolerated dose.

It was not until 2016, when it was published that brequinar is a strong inducer of differentiation in acute myeloid leukemia cells, that this small molecule was brought back into the limelight [32]. Following up these studies, Bayer has started clinical trials on myeloid leukemia patients with a new and extremely potent DHODH inhibitor named BAY 2402234 [97] and other companies have followed this path (https://clinicaltrials.gov/ct2/results?cond=&term=dhodh&cntry=&state=&city=&dist=). Most interestingly, brequinar is active against pancreatic cancer in xenograft studies [109, 110], suggesting a new way to target KRAS mutant tumors and to overcome resistance to Raf, MEK, and ERK inhibitors.

A way to increase the efficacy of DHODH inhibitors against cancer cells is to combine them with other agents. For example, inhibition of DHODH leads to activation of the p53 tumor suppressor and synergizes with inhibitors of p53 degradation (mdm2 inhibitors) to kill cancer cells [33]. One explanation for this synergy between DHODH inhibitors and mdm2 inhibitors is that if cancer cells treated with DHODH inhibitors accumulate in S phase, releasing p53 from mdm2 at this vulnerable stage of the cell cycle may promote cell death. Another interesting feature with regard to the relationship between DHODH and p53 is that a large proportion of small molecules identified through a cell-based screen as activators of p53 can inhibit DHODH [33].

## UMPS inhibitors

Pyrazofurin is an inhibitor of UMPS that acts as a nucleoside analogue and blocks the orotidine 5′-monophosphate decarboxylase activity of UMPS [111]. Unfortunately, there are several difficulties in pursuing this strategy. First, it was seen that resistance to pyrazofurin was easily achieved and that this nucleoside analogue could convert into a nucleotide and potentially incorporate into nucleic acids and cause mutations [112, 113]. An alternative way to inhibit UMPS is by the use of AICAr (5-aminoimidazole-4-carboxamide-1-b-riboside) whose pro-apoptotic effect, at least in the case of multiple myeloma, is thought to be mediated by the inhibition of UMPS [114]. Although the possibility of discovering other UMPS inhibitors could be considered, it may not prove to be safe in the clinic as loss of UMPS activity causes orotic aciduria [115].

Recently, it has been found that uric acid, a purine nucleotide degradation product abundant in human plasma, can inhibit UMPS [116]. In this regard, it may be of importance when evaluating results from preclinical tests with inhibitors of pyrimidine ribonucleotide metabolism, to remember that mouse plasma and human plasma differ significantly with regard to uric acid levels as these are 10-fold higher in the blood of humans than of mice [116].

## Inhibitors of uridine import

Uridine levels in blood are not negligible [16, 43], and this could debilitate the effects of inhibitors of the de novo synthesis pathway described above. However, many inhibitors of uridine uptake are available and several are used as medicaments. Combining inhibitors of CAD, DHODH, and possibly UMPS with blockers of uridine uptake may be an attractive strategy to increase efficacy.

Uridine uptake inhibitors include dipyridamole, which inhibits ENT1 and ENT2 and is used in the clinic to prevent blood clots [117, 118]. Surprisingly, a number of clinically approved tyrosine kinase inhibitors also block uridine uptake by cells [119]. One of the most potent ones is nilotinib [120], which is used as a Bcr-Abl inhibitor to treat chronic myeloid leukemia. Whether the inhibition of uridine uptake by nilotinib contributes to its therapeutic effect is still unknown. Nitrobenzylmercaptopurine ribonucleoside (NBMPR) has been a valuable pharmacological tool used extensively to characterize the ENT transporters [121, 122].

Since CNTs are known to more effectively transport uridine, they may constitute better targets than ENTs. However, no high affinity CNT inhibitors were available until recently. These include thienopyrimidine 2'-deoxynucleoside and ribonucleoside [43, 123]. Interestingly, the tyrosine kinase inhibitor imatinib, although not very potent (IC_50_ = 2.3 μM), can inhibit CNT2 [120].

The expression levels between ENTs and CNTs vary between tissues and tumor types (CCLE database). For example, CNT1 (SLC28A1) is high in the kidneys, liver, and small intestine and, accordingly, highly expressed in kidney cancers. Therefore, characterizing the specificity of small molecules for each of these nucleoside transporters may help target tumors specifically and predict toxic effects.

## CDA inhibitors

Aside from ENTs and CNTs, another target of the salvage pathway is CDA, which converts cytidine into uridine and therefore, together with the CTP synthetases, regulates the ratio between uracil and cytosine nucleotides. CDA inhibitors include tetrahydrouridine [124], but also a new compound, cedazuridine (E7727), which is currently in clinical trials in combination with decitabine for the treatment of myelodysplastic syndromes and chronic myelomonocytic leukemia [125]. The rationale for this combination is that CDA inhibition will prevent deamination of decitabine and therefore increase its bioavailability. The same principle can be extended to azacitidine and cytarabine, which are also susceptible to deamination by CDA. It might be interesting to test whether altering the salvage pathway with CDA inhibitors works in synergy with inhibitors of de novo pyrimidine ribonucleotide synthesis.

## CTPS I and II inhibitors

CTP synthetase activity, which converts UTP into CTP, may be upregulated in tumors according to a study performed decades ago [126]. CTP synthetases form remarkable structures in cells called cytoophidia due to their snake-like shape that are present in the cytoplasm as well as in the nucleus [127]. In humans, CTPS polymerization increases catalytic activity [128] and CTPS activity may be regulated by post-translational modifications or binding to other factors [129]. CTPS filaments assemble at particular developmental stages (e.g., when there is a high demand for CTP) as well as in response to nutrient stress. Indeed, CTPS filaments form in response to glutamine deprivation and disassemble upon glutamine addition to cells [128, 130]. An intriguing question is why active hCTPS forms polymers? In this regard, it is interesting that in *S. pombe* cells, CTPS cytoophidia are asymmetrically inherited during cell division and in a stochastic fashion [131]. Therefore, agents that affect cytoophidia formation on dividing cells may influence the distribution of this CTP synthetase enzymatic activity between daughter cells and, in this manner, affect the proliferation potential of at least one of the daughter cells. Agents that directly affect CTP synthetases include cyclopentenyl cytosine (CPEC), but whether this is a safe anticancer strategy needs to be established as cardiotoxicity has been reported in a phase I trial [132]. In addition, accumulation of UTP might lead to nucleotide imbalance as well as to high dUTP levels.

## DPYD inhibitors

DPYD is involved in the degradation of uracil, and therefore, its inhibition could lead to an increase in UMP levels (Fig. 1). This enzyme can be inhibited by gimeracil and eniluracil, which were designed to improve the efficacy of 5-fluorouracil by decreasing its breakdown by DPYD. Eniluracil failed in phase III trials, but gimeracil is still in clinical trials (https://clinicaltrials.gov).

One question that could be worth investigating is whether these inhibitors also affect the efficacy of UMP synthesis inhibitors. In principle, inhibition of DPYD would be expected to rescue UMP from degradation and therefore weaken the efficacy of inhibitors of de novo pyrimidine ribonucleotide synthesis. If this occurs, one could speculate that tumors that express high levels of DPYD would be more sensitive to UMP synthesis inhibitors.

## Conclusions

The recent advances in the identification of small molecule modulators of pyrimidine ribonucleotide synthesis, and in particular of small molecule inhibitors of DHODH, have led to a renewed interest in this field of research for the treatment of cancer. If the current clinical trials with DHODH inhibitors on leukemia patients show signs of efficacy and low toxicity, the next challenge will be to find synergistic and safe combinations with other agents and to extend the use of pyrimidine ribonucleotide synthesis inhibitors to patients with solid tumors. Bearing in mind the failure of the CAD inhibitor PALA and the DHODH inhibitor brequinar in clinical trials for solid tumors in the 1990s, it is not unreasonable to postulate that identifying molecular signatures in cancer cells that confer hypersensitivity to depletion of pyrimidine ribonucleotide pools will be crucial. Considering the effects of food intake on uridine levels may also be key to improve efficacy. In addition, given the importance of the immune system in the prevention of cancer, the immunosuppressive effect of ribonucleotide synthesis inhibitors must be taken into account.

## Data Availability

Not applicable

## Abbreviations

ADP: Adenosine 5′-diphosphate
ATP: Adenosine 5′-triphosphate
β-ala: β-Alanine
CAD: Carbamoylphosphate synthetase II, aspartate transcarbamoylase, and dihydroorotase
CDA: Cytidine deaminase
CDP: Cytidine 5′-diphosphate
CDP-PL: CDP-linked phospholipids
CMP: Cytidine 5′-monophosphate
CNT: Concentrative nucleoside transporter
Ctd: Cytidine
CPSII: Carbamoylphosphate synthetase II
CTP: Cytidine 5′-triphosphate
CTPS I: Cytidine 5′-triphosphate synthetase I
CTPS II: Cytidine 5′-triphosphate synthetase II
dCDP: 2′-Deoxycytidine 5′-diphosphate
dCMP: 2′-Deoxycytidine 5′-monophosphate
dCtd: Deoxycytidine
DHOA: Dihydroorotic acid
DHODH: Dihydroorotate dehydrogenase
DPYD: Dihydropyrimidine dehydrogenase
dTMP: 2′-Deoxythymidine-5′-monophosphate
dTTP: 2′-Deoxythymidine-5′-triphosphate
dUMP: 2′-Deoxyuridine 5′-monophosphate
dUrd: Deoxyuridine
dUTP: 2′-Deoxyuridine-5′-triphosphate
ENT: Equilibrative nucleoside transporter
hCTPS I: Human cytidine 5′-triphosphate synthetase I
L-Asp: l-Aspartate
L-Gln: l-Glutamine
MAPK: Mitogen-activated protein kinase
OA: Orotic acid
PALA: *N*-Phosphonacetyl-l-aspartate
PKA: Cyclic adenosine monophosphate-dependent protein kinase
PKC: Protein kinase C
PRPP: Phosphoribosyl pyrophosphate
RNR: Ribonucleotide reductase
Thd: Thymidine
UDP: Uridine 5′-diphosphate
UMP: Uridine 5′-monophosphate
UMPS: Uridine monophosphate synthetase
UTP: Uridine 5′-triphosphate
Ura: Uracil
Urd: Uridine
